# Morphometric Study of the Nutrient Foramen of the Humerus in the Population of Bihar

**DOI:** 10.7759/cureus.32856

**Published:** 2022-12-23

**Authors:** Sanjay Kumar, Sanjeev Kumar Sinha, Md Jawed Akhtar, Binod Kumar, Rajiv Ranjan Sinha, Avanish Kumar

**Affiliations:** 1 Department of Anatomy, Indira Gandhi Institute of Medical Sciences, Patna, IND; 2 Department of Anatomy, Narayan Medical College, Sasaram, IND

**Keywords:** diaphysis, foramen index, nutrient artery, nutrient foramina, humerus

## Abstract

Introduction: Fracture or surgical intervention of fracture of the shaft of the humerus may cause injury to the nutrient artery leading to the nonunion or delayed union of the fracture. It is important to find the number and location of the nutrient artery. So the knowledge regarding the nutrient foramen helps to protect them during any operative procedure of the shaft of the humerus. The main objective of this study is to find out the number, location, and direction of the nutrient foramen of the humerus.

Materials and methods: The study was conducted on 80 dried humeri of unknown gender obtained from Narayan Medical College, Sasaram, Bihar, India, and also from other medical colleges of Bihar. The number, location, and direction of nutrient foramen were observed.

Results: The majority of humeri showed one nutrient foramen, which was found in 91.25%, followed by 3.75% with double foramen and 1.25% with triple foramen. Nutrient foramen was absent in 3.75% of the humerus. The majority (89.02%) of nutrient foramen was found on the anteromedial surface followed by anterolateral (9.76%) and posterior surface (1.22%). The majority of nutrient foramen was found on the middle third (86.58%) of the shaft, followed by 13.42% on the distal third. No nutrient foramen was found on the proximal third of the humerus. All nutrient foramina were directed downward.

Conclusion: The location of the nutrient foramen of the humerus was not constant; it may present on anteromedial, anterolateral, or posterior surfaces. Similarly, it may present on the middle or distal third of the shaft of the humerus. This study will help surgeons planning the surgical intervention of the shaft of the humerus, which will possibly reduce the chances of nonunion or delayed union.

## Introduction

Fractures of long bones are not uncommon in the modern era due to changes in lifestyle and dependency on machinery. The blood supply of long bones plays an important role in the healing of fractures [1,2]. Long bones derive their blood supply through the nutrient, periosteal, metaphyseal, and epiphyseal arteries. The medulla and inner half of the cortex of the shaft of long bones are supplied by the nutrient artery. In contrast, the outer cortex of the shaft and metaphysis are supplied by periosteal and metaphyseal arteries [3]. The nutrient artery enters the shaft through the nutrient foramen leading into the nutrient canal [4]. The site of entry of the nutrient artery is almost always constant and directed away from the growing end [5]. A nutrient foramen is located at the anteromedial surface in the middle one-third of the shaft of the humerus [6]. The nutrient artery enters through the nutrient foramen, which is a branch of the brachial artery [7]. It is the main source of blood supply to the humerus and is also important during the active growth period of the fetus and during the early phase of ossification [8]. The nutrient foramen of the humerus is directed toward the elbow. The knowledge of the location of the nutrient foramen is important in operative procedures to preserve circulation [9-11]. The vascular system of bone is closely related to fracture healing and hematogenic osteomyelitis [12]. Detailed knowledge about the blood supply of long bones is important in the development of new transplantation and resection techniques in orthopedics. This study aimed to find out the number and location of the nutrient foramen in relation to different surfaces, and the site of the nutrient foramen in relation to different segments and directions of the nutrient foramen of the humerus in the population of Bihar. The aim of this study was to determine the number, location, site, and direction of the nutrient foramen in the population of Bihar.

## Materials and methods

The present study, an analytical type of observational study, was conducted on 80 (40 right and 40 left) dry humeri of unknown sexes obtained from Narayan Medical College, Sasaram, Bihar, and also from other medical colleges of Bihar, India during the period from May 22, 2019, to May 19, 2021. Ethical permission was taken from Narayan Medical College and Hospital (letter no.: NMCH/IEC No/2019/9). Bones that were damaged and had healed fractures, congenital anomalies, and significant pathological changes were excluded from this study. Based on the above criteria, 16 bones were excluded. Side determination was done for all humeri. The nutrient foramina were distinguished by the presence of a well-marked groove leading to the foramen. All humeri were studied for the number, location, site, and direction of the nutrient foramen. Nutrient foramen was observed with the help of magnifying glass. If the humerus has more than two nutrient foramina, the foramen that is larger in size is called the dominant foramen, and the other is called the secondary foramen.

The position of the nutrient foramen was determined by the foraminal index (FI) using the following formula [13]: FI = DNF/TL x 100. Where DNF is the distance of nutrient foramen from the most proximal part of the humerus and TL is the total length of the humerus.

The position of the nutrient foramen was divided into three types according to FI: type 1 = FI < 33.33, where the foramen is located in the proximal third of the humerus; type 2 = FI between 33.33 and 66.66, where the foramen is located in the middle third of the humerus; type 3 = FI > 66.66, where the foramen is located in the distal third of the humerus.

The total length of humeri was measured by an osteometric board in centimeters. The distance of nutrient foramen from the most proximal point of the humerus was measured by a digital vernier caliper in centimeters. All observations were tabulated and statistically analyzed using a Microsoft Excel worksheet (Microsoft Corporation, Redmond, WA).

## Results

**Figure 1:**
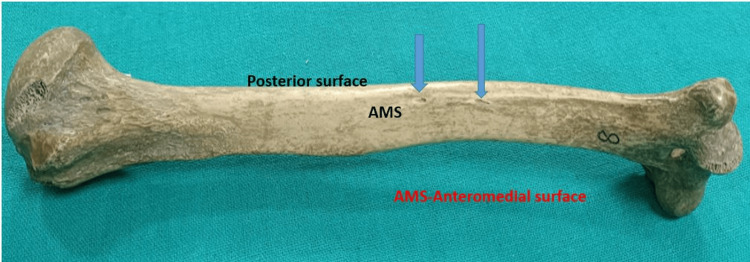
Two nutrient foramina on the anteromedial surface. One is on the middle third and the other one is on the distal third

**Figure 2:**
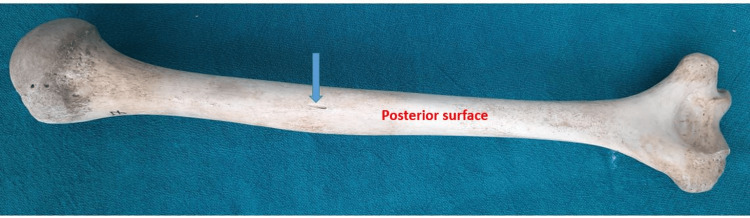
Nutrient foramen on the posterior surface

The nutrient foramina were absent in three (3.75%) of the humerus. Single nutrient foramen was observed in 37 (46.25%) of the right humerus, 36 (45%) of the left humerus, and 73 (91.25%) of the total humerus. Two nutrient foramen were observed in two (2.50%) of the right humerus, one (1.25%) of the left humerus, and three (3.75%) of the total humerus. Three nutrient foramen were observed only in one (1.25%) of the left humerus (Table 1 and Figures 1, 2).

**Table 1:** Incidence of the number of the nutrient foramen of the humerus

	Right		Left		Total	
No. of nutrient foramen	No. of humerus (n = 40)	Percentage (%)	No. of humerus (n = 40)	Percentage (%)	No. of humerus (n = 80)	Percentage (%)
-	1	1.25%	2	2.50%	3	3.75%
1	37	46.25%	36	45%	73	91.25%
2	2	2.50%	1	1.25%	3	3.75%
3	-	-	1	1.25%	1	1.25%
	40		40		80	

It has been observed that a total of 82 nutrient foramina were observed and they were present on anteromedial, anterolateral, and posterior surfaces. Out of 82 nutrient foramina, 89.02% were present on the anteromedial surface, 87.8% on the right side, and 90.02% on the left side. Of the nutrient foramen, 9.76% were found on the anterolateral surface, out of which 9.76% were on the right and 9.76% were on the left side. The posterior surface had 1.22% of the nutrient foramen, out of which 2.44% were on the left side. All nutrient foramina were directed downward (Table 2 and Figures 1, 2).

**Table 2:** Location of the nutrient foramen in relation to different surfaces of the humerus

Surface	Right		Left		Total	
	Number (n = 41)	Percentage (%)	Number (n = 41)	Percentage (%)	Number (n = 82)	Percentage (%)
Anteromedial	36	87.80	37	90.24	73	89.02
Posterior	1	2.44	-	-	1	1.22
Anterolateral	4	9.76	4	9.76	8	9.76
Total	41	100	41	100	82	100

The incidence of nutrient foramina in relation to different parts of the shaft of the humerus was described in the proximal one-third, middle one-third, and distal one-third. Out of a total of 82 nutrient foramen, the maximum number was observed in the middle one-third of the shaft (86.58%), followed by the distal third (13.42%). No nutrient foramen was found on the proximal one-third of the shaft. It was observed that all nutrient foramina were directed downward and toward the lower end of the humerus, i.e., away from the growing end (Tables 3, 4 and Figure 1).

**Table 3:** Site of the nutrient foramen in relation to different segments of the humerus

Situation	Right		Left		Total	
	Number (n = 41)	Percentage (%)	Number (n = 41)	Percentage (%)	Number (n = 82)	Percentage (%)
Proximal 1/3	-	-	-	-	-	-
Middle 1/3	35	85.36%	36	87.80%	71	86.58%
Distal 1/3	6	14.63%	5	12.19%	11	13.42%
Total	41	100%	41	100%	82	100%

**Table 4:** Length of the humerus, the distance of nutrient foramina from the proximal end, and the foraminal index The total length of the humeri was found to be 30.07 ± 1.89 cm in the right, 30.15 ± 2.02 cm in the left, and 30.11 ± 1.94 cm in total humeri. The mean distance from the proximal end of the humerus to the nutrient foramen was found to be 16.63 ± 1.23 cm in the right, 16.66 ± 1.22 cm in the left, and 16.65 ± 1.22 cm in total humeri. The foraminal index was found to be 55.53% in the right, 55.54% in the left, and 55.53% in total humeri.

Parameter	Right (n = 40)	Left (n = 40)	Total (n = 80)
Mean total length (cm)	30.07 ± 1.89	30.15 ± 2.02	30.11 ± 1.94
Distance of nutrient foramina from the proximal end (cm)	16.63 ± 1.23	16.66 ± 1.22	16.65 ± 1.22
Foraminal index	55.53%	55.54%	55.53%

## Discussion

A comparison of the incidence of the number of the nutrient foramen is present in Table 5.

**Table 5:** Comparison of the incidence of the number of the nutrient foramen

Authors	No. of humerus (n)	Single nutrient foramen	Two nutrient foramen	Three nutrient foramen	Absent nutrient foramen
Carrol et al. (1963) [14]	71	48 (68%)	20 (28%)	3 (4%)	-
Halagatti and Rangasubhe (2012) [15]	200	161 (80.5%)	35 (17.5%)	4 (2%)	-
Joshi et al. (2011) [16]	200	126 (63%)	66 (33%)	8 (4%)	-
Laing et al. (1956) [2]	30	28 (93%)	2 (7%)	-	-
Chandrasekaran et al. (2013) [3]	258	198 (76.74%)	53 (20.54%)	7 (2.71%)	-
Mansur et al. (2016) [17]	253	154 (60.87%)	73 (28.85%)	16 (6.32%)	-
Asharani and Ningaiah (2016) [18]	120	104 (87%)	20 (11%)	-	2 (2%)
Pankaj et al. (2017) [19]	350	283 (80.86%)	47 (13.42%)	01 (0.29%)	19 (5.43%)
Ali (2021) [20]	250	210 (84%)	35 (14%)	-	5 (2%)
Ramya Sree et al. (2019) [13]	218	169 (81.19%)	40 (18.35%)	1 (0.45%)	8 (3.67%)
Arfan et al. (2022) [4]	86	52 (60.40%)	25 (29.06%)	5 (5.81%)	4 (4.65%)
Bhatnagar et al. (2014) [21]	70	63 (90%)	5 (7.14%)	1 (1.43%)	1 (1.43%)
Present study (2022)	80	73 (91.25%)	3 (3.75%)	1 (1.25%)	3 (3.75%)

The present study showed that single nutrient foramen was present in 91.25% of humeri. A similar finding was seen in studies by Laing (93%) [2] and Bhatnagar et al. (90%) [21]. Many studies reported a lower incidence of single nutrient foramen [4,16]. Joshi et al. [16] and Arfan et al. [4] reported single nutrient foramen only in 63% and 60.40% of humerus, respectively. The present study showed that the prevalence of double nutrient foramen was found in 3.75% of humeri, which was very similar to the study done by Laing (7%) [2] and Bhatnagar et al. (7.14%) [21]. Joshi et al. [16] found a higher incidence of a double nutrient foramen in 33% of humeri. Almost all authors observed the presence of triple nutrient foramina in humeri [14-16]. The present study observed that triple nutrient foramen was found in 1.25% of humeri, which was very close to studies done by Halagatti and Rangasubhe (2%) [15] and Bhatnagar et al. (1.43%) [21]. In this study, it has been observed that 3.75% of humeri did not have nutrient foramen, which was very similar to the study done by Ramya Sree et al. [13], who reported that in such cases, 3.67% of humeri are supplied by periosteal arteries (Table 5) [22].

The nutrient foramen is located on the anteromedial surface of the shaft of the humerus close to the medial border; however, its location may vary. In the present study, 89.02% of foramina were situated on the anteromedial surface, which was in accordance with the findings of Chandrasekaran et al. (89.92%) [3] and Mansur et al. (88.86%) [17]. In contrast to this, a study done in Pakistan by Khan et al. [23] reported a higher incidence (96%) of nutrient foramina situated on the anteromedial surface (Table 6).

**Table 6:** Comparison of the location of the nutrient foramen in relation to surface

Author	No. of humerus (n)	Anteromedial surface	Posterior surface	Anterolateral surface
Mansur et al. (2016) [17]	253	88.86%	6.52%	4.62%
Chandrasekaran et al. (2013) [3]	258	89.92%	8.53%	1.55%
Yaseen et al. (2014) [12]	100	88.50%	8.53%	3.50%
Khan et al. (2014) [23]	75	96%	2.67%	1.33%
Present study (2022)	80	89.02%	1.22%	9.76%

In the present study, 86.58% of nutrient foramina were located in the middle one-third of humeri followed by distal one-third in 13.42%. No nutrient foramina were found on the proximal one-third of the humerus. This finding is in agreement with that of Chandrasekaran et al. [3] and Yaseen et al. [12] (Table 7).

**Table 7:** Comparison of the site of the nutrient foramen in relation to the segment

Author	No. of humerus (n)	Proximal 1/3^rd^	Middle 1/3^rd^	Distal 1/3^rd^
Pankaj et al. (2017) [19]	350	0.53%	97.63%	1.84%
Mansur et al. (2016) [17]	253	0.54%	94.84%	4.62%
Arfan et al. (2022) [4]	86	4.87%	91.46%	3.65%
Yaseen et al. (2014) [12]	100	-	89%	11%
Chandrasekaran et al. (2013) [3]	258	-	86.43%	13.57%
Present study (2022)	80	-	86.58%	13.42%

The nutrient artery is the main source of blood during the active growth of long bones. The correlation of the direction of the nutrient canal with the mode of ossification and growth of bone was first described by Berard (1835) [24]. The humerus also receives blood from metaphyseal and periosteal arteries, which branch from the axillary and brachial arteries. The knowledge of variations of the nutrient foramen is important for orthopedic surgeons who undertake the open reduction of fracture to avoid injury of nutrient artery thus decreasing the chances of delayed union or nonunion of fracture [16]. The intact blood supply of bone is very important for the healing of a fractured bone [25]. It is well understood that delayed union or nonunion of fracture of bone occurs due to lack of arterial supply [26].

## Conclusions

This study concludes that the nutrient foramen of the humerus is not only confined to the anteromedial surface but it may also be found on anterolateral and posterior surfaces. Similarly, nutrient foramen was found on the middle and distal third of the shaft of the humerus. Most of the humerus had single nutrient foramen but two or three nutrient foramina were also found in the humerus. So, the knowledge of the number, location, site, and direction of nutrient foramen will be helpful for orthopedic surgeons to avoid this area during internal fixation, fracture repair, bone graft, joint replacement therapy, and vascularized bone microsurgery to minimize the chances of damage to the nutrient artery. Damage to the nutrient artery may lead to the nonunion or delayed union of bone following fracture of the shaft of the humerus.
